# Analysis of erythrocyte deformation characteristics based on dual-angle Mueller matrix measurement

**DOI:** 10.1007/s12200-025-00166-2

**Published:** 2025-12-03

**Authors:** Shuan Yao, Caizhong Guan, Nan Zeng, Honghui He

**Affiliations:** 1 Tsinghua Shenzhen International Graduate School, Tsinghua University, Shenzhen, 518055 China; 2 Guangdong Research Center of Polarization Imaging and Measurement Engineering Technology, Shenzhen Key Laboratory for Minimal Invasive Medical Technologies, Institute of Optical Imaging and Sensing, Shenzhen, 518055 China

**Keywords:** Polarization, Red blood cell, Cell deformation, Single-cell analysis

## Abstract

Red blood cells (RBCs) are vital components of human blood, and their morphological abnormalities serve as reliable indicators of various disease pathophysiologies. As a novel label-free optical technique, Mueller matrix (MM) polarimetry is gaining recognition for its value in disease diagnosis and pathological analysis. In this study, we integrate a dual-angle MM measurement system with single-cell polarized light scattering modeling to establish specific polarization feature parameters (PFPs) characterizing cellular microphysical properties. The PFPs quantitatively describe morphological and optical changes in individual RBCs undergoing complex deformations. Experimental results demonstrate that PFPs can effectively distinguish differences in size, shape, refractive index, and surface spicules between deformed and normal RBCs. Moreover, by incorporating PFPs into a Random Forest classifier, we accurately quantify the proportion of abnormal RBCs in mixed suspensions. This study confirms the capability of polarization measurement for label-free, high-throughput analysis of RBC microphysical properties at the single-cell level.

**Graphic abstract:**

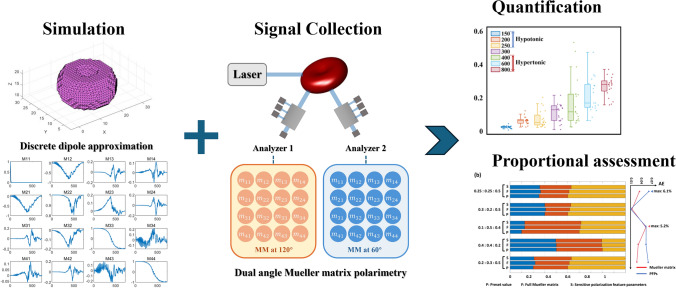

## Introduction

Red blood cells (RBCs) are key functional cells in the human body, playing essential roles in oxygen transport [1], acid–base balance [2], and immunity [3]. Extensive research has demonstrated that alterations in RBC morphology are intricately linked to the pathophysiology of various diseases, including diabetes [4–6], malaria [7–9], hereditary blood disorders [10–12], and vascular inflammatory diseases [13]. Apart from pathological factors, RBCs stored in vitro undergo morphological and mechanical changes during aging, increasing the risk of complications in transfusion recipients [14]. Therefore, non-invasive and accurate assessment of RBC physical property changes induced by abnormal deformation is crucial for diagnosing and prognosing related diseases.

Currently, RBC morphology analysis primarily utilizes optical microscopy [15], electron microscopy [16], and super-resolution microscopy (e.g., Stimulated Emission Depletion microscopy and Stochastic Optical Reconstruction Microscopy) [17]. Among these, optical microscopy is the clinical gold standard but is limited by operator-dependent subjectivity, poor reproducibility, and the requirement for fluorescence staining. Additionally, it exhibits inadequate sensitivity to subtle changes in individual RBCs. Electron microscopy and super-resolution microscopy visualize RBC morphology and structure at the nanoscale. However, they are costly, operationally complex, slow, and often require time-consuming fluorescent staining.

Complementing these imaging approaches, several label-free techniques, including quantitative phase imaging (QPI) [18], atomic force microscopy (AFM) [19], Raman scattering [20], and elastic light scattering [21], can also be used for RBC analysis. While QPI and AFM enable high-resolution and multi-dimensional imaging, their limited fields of view and complex data processing hinder high-throughput detection. Unlike Raman scattering, which extracts information on molecular vibrational and chemical composition, elastic light scattering captures the angular distribution of scattered light, reflecting the size and shape of RBCs. It can also be combined with flow cytometry to achieve high-throughput analysis.

As a fundamental property of light, polarization can provide rich information on cell morphology and microstructure. Polarization measurement offers great potential for cancer cytodiagnosis and viral infection analysis, owing to its non-invasiveness, high sensitivity, and specificity [22–24]. Our previous studies have demonstrated the effectiveness of the Mueller matrix characterization in analyzing RBC suspensions [25]. However, this population-level analysis approach has limitations in revealing changes within individual cells. Therefore, this study focuses on the polarization analysis for individual RBCs and the correlation between polarization scattering characteristics and physical properties.

For theoretical cell modeling, the discrete dipole approximation (DDA) can simulate the polarization characteristics of individual RBCs with arbitrary shapes in the scattering space [26]. Owing to their relatively simple internal structure and thin membrane, RBCs can be modeled as scatterers with a constant refractive index [27]. By adjusting parameters such as size, shape, and refractive index, the DDA method accurately describes the light polarization response of RBCs undergoing complex deformations, providing extensive theoretical support for experimental findings.

In this study, we develop a method combining a dual-angle Mueller matrix polarimeter (DMMP) with polarized light scattering simulation to extract polarization feature parameters (PFPs). These PFPs identify and characterize the physical properties associated with complex deformations in individual RBCs. We first employ the DDA method to simulate the polarization responses of RBCs undergoing three typical deformation modes: spherocyte (or elliptocyte) formation, discocyte flattening, and echinocyte formation. Subsequently, specific PFPs are extracted to quantify key deformation features, including equivalent diameter, refractive index, concavity, sphericity, and surface spiculation. Further experiments involving RBCs subjected to osmotic, oxidative, and acid–base stress validate the PFPs’ capabilities for cell characterization, applicability, and interpretability. Additionally, by combining PFPs with a random forest classification algorithm, we accurately determine the proportion of normal and abnormal RBCs in cell suspensions. This study demonstrates the potential of DMMP technology and its derived PFPs in accurately identifying and quantifying red blood cell deformation at the single-cell level.

## Methods and materials

### Sample preparation

We use mouse RBC suspensions (4% hematocrit; HongQuan Biological Technology Co., Ltd., Guangzhou, China) as experimental samples.

To simulate RBC morphological abnormalities under pathological conditions, we apply three stress types: osmotic, oxidative, and acid–base. Osmotic stress simulates hydration abnormalities caused by abnormal plasma osmolarity and hereditary anemia [28]. Oxidative stress simulates cell damage from increased intracellular reactive oxygen species (ROS) and weakened antioxidant defenses under disease conditions [29]. Acid–base stress can simulate RBC morphological changes induced by systemic acid–base imbalances (e.g., acidosis or alkalosis) [30]. The experimental procedures for each stress type are detailed below:

#### Osmotic stress

We dilute 100 μL of the initial RBC suspension in 80 mL of NaCl solution at varying osmolalities. Specifically, the osmolality between 150 and 800 mOsm is adjusted by preparing NaCl solutions at concentrations of 75 mmol, 100 mmol, 125 mmol, 150 mmol, 200 mmol, 300 mmol, and 400 mmol, corresponding to osmolalities of 150 mOsm, 200 mOsm, 250 mOsm, 300 mOsm, 400 mOsm, 600 mOsm, and 800 mOsm, respectively.

#### Oxidative stress

The oxidative stress condition is induced by H_2_O_2_. A 30 mmol/L H_2_O_2_ solution is added to the RBC suspension for the experimental group. The control group receives an equal volume of isotonic phosphate-buffered saline (PBS; pH 7.4). Following incubation of both groups at 37 °C for 1 h, we perform polarization analysis.

#### Acid–base stress

RBC suspensions are mixed with isotonic PBS at pH 6.8 (acidic) or pH 8.0 (alkaline) at a 1:50 volume ratio to create the experimental groups. The control group consists of RBC suspensions mixed with isotonic PBS (pH 7.4) at the same ratio.

To further assess DMMP’s capacity to distinguish abnormal RBCs in blood, we prepare mixtures containing RBCs subjected to osmotic stress (generating spherocytes and echinocytes) and normal RBCs. Cells are mixed at the following volume ratios (spherocytes: echinocytes: normal): 0.25:0.25:0.5, 0.3:0.2:0.5, 0.1:0.5:0.4, 0.4:0.4:0.2, and 0.2:0.3:0.5, then diluted with PBS. To maintain stable morphology, the generated spherocytes and echinocytes are fixed with a neutral PBS containing 1% glutaraldehyde at a 1:50 volume ratio. The fixed RBCs are then washed three times with PBS and resuspended in the original medium following centrifugation. All experiments are performed at 25 °C.

### Experimental setup

**Fig. 1:**
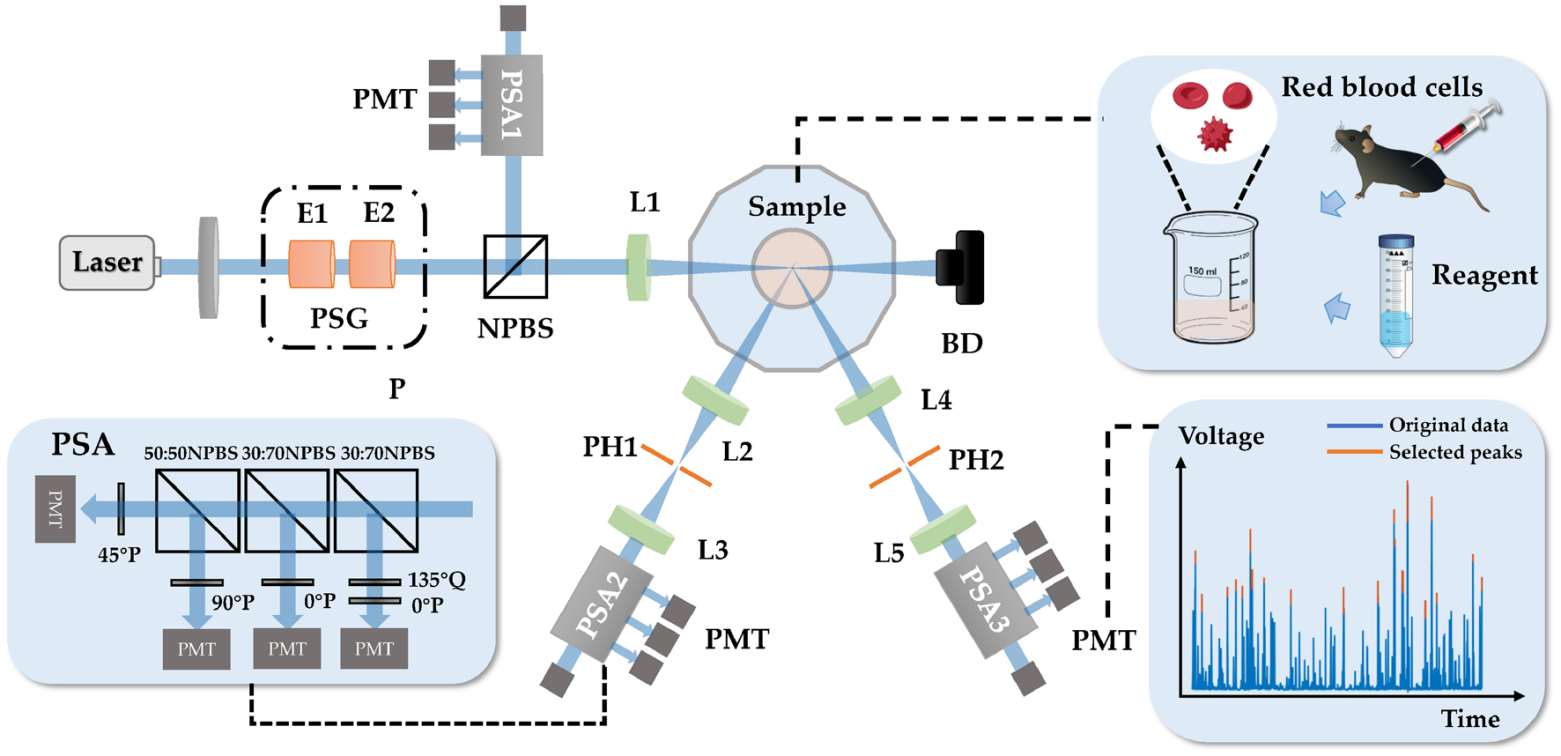
Schematic diagram of DMMP. PSG: polarization state generator; E1, E2, electro-optic modulator; NPBS: Non-polarizing beam splitter cube; L1, L2, L3, L4, L5: lenses; Sample: mouse RBC suspensions prepared by mixing with various reagents; BD: beam dump; PH1, PH2: pinhole; PSA: polarization state analyzer; P: polarizer; Q: quarter-wave plate; PMT: photomultiplier tube

In this study, DMMP is employed to analyze the polarization scattering signals from individual RBCs in suspension under various experimental conditions. As illustrated in Fig. 1, the system comprises three components: an illumination path, a sample chamber, and two detection paths.

A 473 nm solid-state laser (300 mW) serves as the illumination source. During measurement, the PSG, comprising a polarizer and two electro-optic modulators, modulates incident light to generate arbitrary states of polarization (SOPs) with a switching speed up to 25 kHz. The modulated light is then split by an NPBS into two paths: one focused by lens L1 to form a sub-100 μm spot on the sample chamber, and the other directed to a four-quadrant PSA for reference.

The sample chamber contains a dodecahedral glass beaker filled with refractive-index-matched distilled water to minimize interface reflections. Centrally positioned RBC suspension is uniformly stirred with a magnetic stirrer. During the experiment, random RBCs traversing the detection area generate scattered light pulses through instantaneous light-cell interactions.

Scattered light at 60° (forward) and 120° (backward) angles is collected simultaneously. Lenses L2/L4 refocus the light through pinholes (PH1/PH2) before collimation by lenses L3/L5. Each pinhole is conjugate to the illumination focal plane and object-space lens L1. The pinhole size is adjusted to constrain the scattering volume, enabling single-cell detection at concentrations < 10^5^ cells per mL.

A four-quadrant PSA synchronously measures incident and scattered SOPs. The PSA contains two 30:70 NPBS and one 50:50 NPBS that split the beam into four paths. Each path processes specific polarization components: a 45° polarizer for 45° linear polarization, a 90° polarizer for horizontal polarization, a 0° polarizer for vertical polarization, and a 135° quarter-wave plate with a 0° polarizer for left-handed circular polarization. PMTs convert optical signals to voltage signals $[s_{0} ,s_{45} ,s_{90} ,s_{L} ]^{\text{T}}$, from which the scattered-light Stokes vector is derived via Eq. (1).

1 $$\overrightarrow {{\varvec{S}}}_{{{\varvec{out}}}} = \left[ {\begin{array}{*{20}c} I \\ Q \\ U \\ V \\ \end{array} } \right] = \left[ {\begin{array}{*{20}c} {s_{0} + s_{90} } \\ {s_{0} - s_{90} } \\ {2s_{45} - s_{0} - s_{90} } \\ {s_{0} + s_{90} - 2s_{L} } \\ \end{array} } \right].$$

$$\overset{\lower0.5em\hbox{$\smash{\scriptscriptstyle\rightharpoonup}$}}{{\varvec{S}}}_{{{\varvec{out}}}}$$ denotes the Stokes vector of the signal light. *I*, *Q*, *U*, and *V* are four components of the Stokes vector, where *I* is the total light intensity, *Q* is the intensity difference between horizontal and vertical linear polarization, *U* is the intensity difference between 45° and 135° linear polarization, and *V* is the intensity difference between right and left circular polarization.

### Data analysis

#### MM acquisition and calibration

Rapid modulation of electro-optic crystals combined with synchronous PSA enables efficient generation of multiple incident and scattered SOP combinations. These combinations cover a broad region on the Poincaré sphere, ensuring high-accuracy MM calculation. The MMs at two scattering angles, obtained by Eq. (2), fully characterize the polarization properties of the RBCs.

2 $${\varvec{MM}} = \left[ {\begin{array}{*{20}c} {M_{11} } & {M_{12} } & {M_{13} } & {M_{14} } \\ {M_{21} } & {M_{22} } & {M_{23} } & {M_{24} } \\ {M_{31} } & {M_{32} } & {M_{33} } & {M_{34} } \\ {M_{41} } & {M_{42} } & {M_{43} } & {M_{44} } \\ \end{array} } \right] = \overrightarrow {{\varvec{S}}}_{{{\varvec{out}}}} \cdot \overrightarrow {{\varvec{S}}}_{{{\varvec{in}}}}^{\user2{ + }},$$

where $\overrightarrow {{\varvec{S}}}_{in}$ denotes the incident Stokes vector, and $\overrightarrow {{\varvec{S}}}_{{{\varvec{in}}}}^{\user2{ + }}$ is the Penrose–Moore inverse matrix of $\overrightarrow {{\varvec{S}}}_{in}$.

To correct system-induced errors, a calibration algorithm using standard polystyrene microspheres is applied [31]. The system error model is defined as Eq. (3).

3 $${\varvec{MM}}_{{\varvec{r}}} = {\varvec{M}}_{{\varvec{p}}} \cdot {\varvec{MM}}_{{\varvec{m}}} - {\varvec{M}}_{{\varvec{s}}},$$

where ${\varvec{MM}}_{{\varvec{r}}}$ is the reference MM of standard particles calculated by Mie theory. ${\varvec{MM}}_{{\varvec{m}}}$ is the measured MM. ${\varvec{M}}_{{\varvec{p}}}$ represents polarization bias from optical components, and ${\varvec{M}}_{{\varvec{s}}}$ accounts for non-polarization errors.

#### Random forest algorithm

Random Forest (RF) is an ensemble learning algorithm based on the bagging strategy [32]. During training, it constructs multiple decision trees. Each tree is trained on a bootstrap sample drawn with replacement from the original training data set. The tree grows by recursively partitioning its bootstrap sample. At each node, a random subset of features is selected as candidate splitting variables. For every candidate feature, all possible split thresholds are evaluated using the node's samples. In classification tasks, the Gini impurity criterion quantifies the purity gain for each potential split. The feature–threshold combination that maximizes the reduction in Gini impurity is chosen to partition the data into child nodes. This process iterates recursively until a stopping criterion is met—typically when a predefined maximum depth is reached or the node contains fewer samples than a specified minimum. Once trained, individual trees predict the class of new inputs, and the final RF output is determined by majority vote across the entire ensemble.

The training approach using random data subsets and random features effectively reduces the risk of overfitting and enhances the model’s robustness against noise and outliers. Therefore, RF is particularly suitable for analyzing high-dimensional polarization data.

### Cell optical model and simulation

**Fig. 2:**
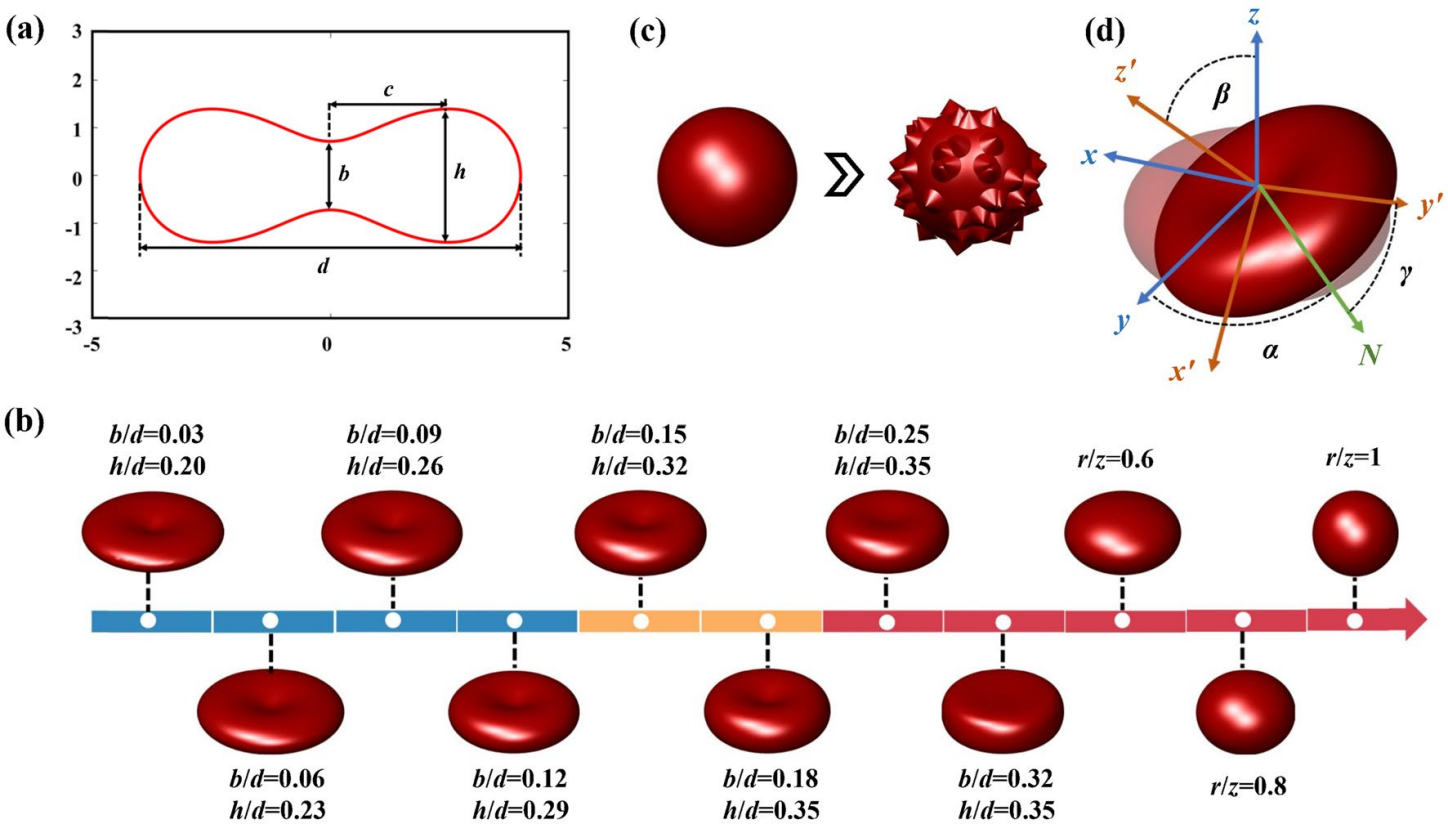
**a** Cross-section of the standard RBC model with four key parameters. **b** Shape parameter configurations for simulating changes in RBC concavity and sphericity. **c** Simulation of RBC spiculated deformation. **d** RBC orientation configurations in the Euler coordinate system

As shown in Fig. 2a, normal RBCs exhibit a biconcave disk morphology defined by four parameters: diameter *d*, central thickness *b*, maximum thickness *h*, and minimum diameter *c*. In the Cartesian coordinate system, the biconcave profile is modeled by Eq. (4), where $r = \sqrt {x^{2} + y^{2} }$ and coefficients *S*, *P*, *Q*, and *R* are determined by the above four parameters [33].

4 $$r^{4} + 2Sr^{2} z^{2} + z^{4} + Pr^{2} + Qz^{2} + R = 0.$$

We employ the DDA method to simulate how RBC size and morphology affect polarization scattering. Each RBC volume is discretized into *N* dipoles on a cubic grid with an interval of $d_{s}$. RBCs are modeled in saline or PBS solutions (refractive index 1.33), with an incident wavelength of 0.472 μm matching experimental conditions. Typical deformation modes, including spherization, flattening, and spiculation, are simulated by varying equivalent diameter, refractive index, concavity, sphericity, and surface spiculation. The details are as follows.

#### Equivalent diameter

The equivalent diameter is defined as the diameter of a sphere with volume equal to that of an RBC. Based on morphological data, the typical RBC diameter ranges from 6 to 9 μm [34]. Therefore, we fix the shape ratios ($b/d = 0.18$, $c/d = 0.62$, and $h/d = 0.35$), while varying *d* from 5 to 9 μm in 0.4 μm increments. The real part of the relative refractive index is set to 1.053 [35], and the imaginary part to 10^−4^ [36].

#### Refractive index

The refractive index depends primarily on intracellular hemoglobin concentration. In vitro, hemoglobin content per RBC remains constant. However, deformation-induced volume changes alter hemoglobin concentration, thereby modulating the refractive index. For each simulated diameter, the real part of the relative refractive index varies from 1.037 to 1.067 in steps of 0.003, with the imaginary part fixed.

#### Shape

We vary the relative maximum thickness $h/d$ and relative central thickness $b/d$ while holding equivalent diameter and refractive index constant. The model increases edge curvature while reducing central concavity. Consequently, the cell morphology transitions from a biconcave disc to a sphere, as illustrated in Fig. 2b.

#### Spiculation

To investigate spiculation effects on RBC polarization properties, conical protrusions are added to random surface regions of a spherical model, forming spiculated morphologies, as shown in Fig. 2c. Protrusion amplitude increases from *d*/20 to *d*/10, where the denominator decreases in steps of 1.

Unlike spherical models, RBC orientation induces polarization scattering anisotropy. To mimic free-floating RBCs, we perform full-space orientation sampling that computes the MM per orientation and averages the results to obtain realistic scattering properties. In Fig. 2d, orientation is defined by Euler angles *α*, *γ* ∈ [0°, 360°], and *β* ∈ [0°, 180°]. For centrally symmetric models (e.g., biconcave disc, ellipsoid), orientation averaging simplifies to sampling *β* only when light is incident along the axis of symmetry. We randomly sample 30 orientations over *β* ∈ [0°, 180°]. However, a full-space orientation calculation is required for spiculated RBC models without symmetry. Due to computational constraints, we randomly select five values per Euler angle to ensure the convergence of the orientation averaging calculations. Consequently, the final orientation average uses 125 scattering directions.

All DDA simulations use our GPU-accelerated Nvidia MATLAB Discrete Dipole Approximation (NMDDA) algorithm [37], and validation against Mie theory for spherical particles shows errors < 0.01 [38]. In addition, the dipole spacing is set as $\left| {mkd_{s} } \right| \approx 0.45$ to balance computational efficiency and accuracy.

## Results and discussion

### Analysis of theoretical MM of RBC models

**Fig. 3:**
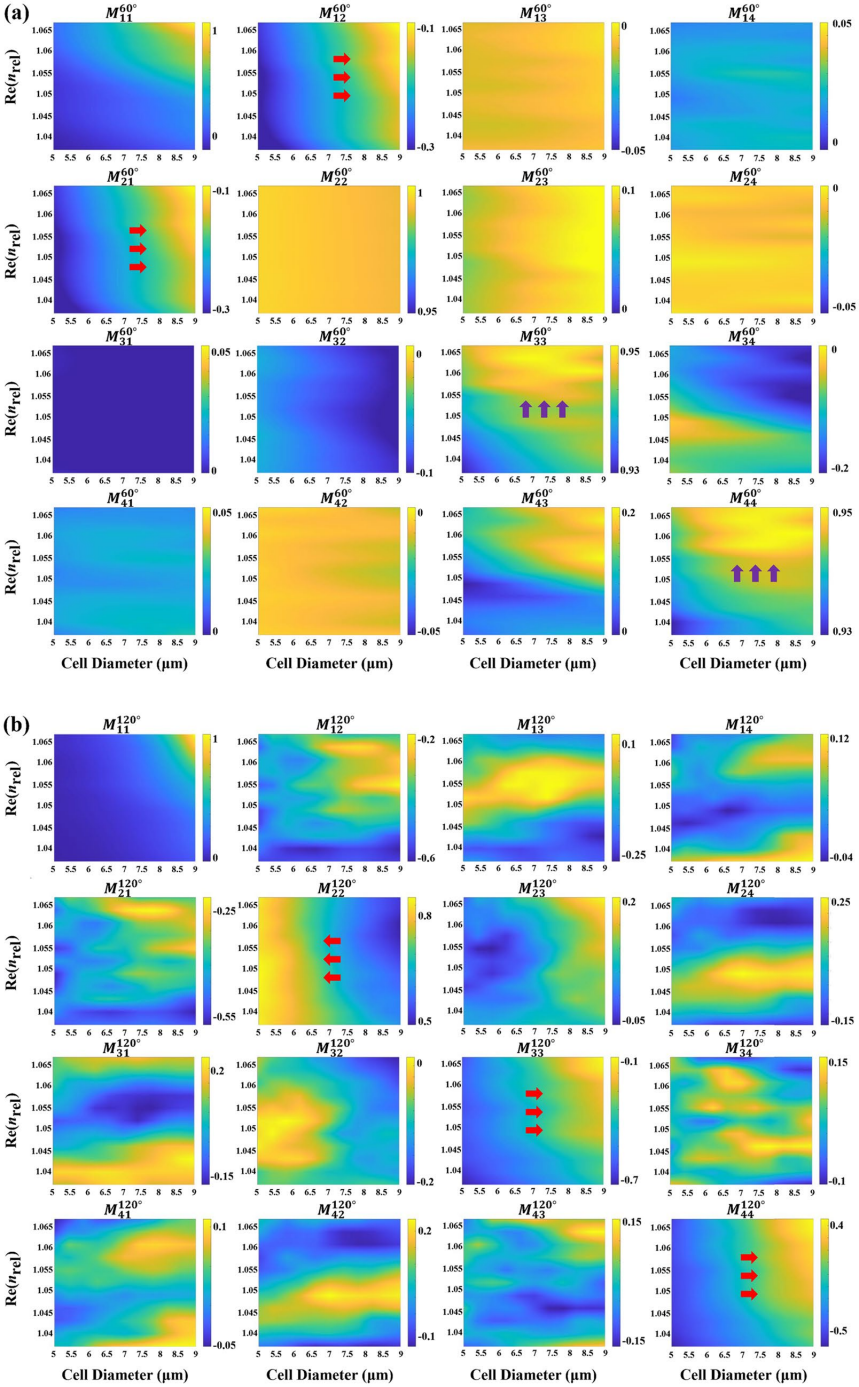
The orientation-averaged MM results for a standard biconcave RBC model with different cell diameters and real parts of the refractive index at **a** 60° and **b** 120°. The $M_{11}^{{60^{^\circ } }}$ and $M_{11}^{{120^{^\circ } }}$ are normalized relative to their values at 0°, while all other MM elements are normalized relative to $M_{11}$ at the corresponding angle

Following the parameters in Sect. 2.4, we simulate standard biconcave RBCs with varying diameters and refractive indices. For each model, we compute angle-resolved MM distribution in the scattering plane and extract values at 60° and 120° to match experimental collection angles. The effects of changes in cell diameter and refractive index on RBC polarization scattering are presented in Fig. 3.

RBC model symmetry enforces reciprocity of non-diagonal elements of MM: $M_{ij} = \pm M_{ji}$ [39], which is evident by simulation results at 60° and 120°. As shown in Fig. 3, red arrows highlight MM elements monotonic in diameter for a fixed refractive index, such as $M_{12}^{{60^{^\circ } }}$, $M_{21}^{{60^{^\circ } }}$, $M_{22}^{{120^{^\circ } }}$, $M_{33}^{{120^{^\circ } }}$ and $M_{44}^{{120^{^\circ } }}$. Specifically, $M_{12}^{{60^{^\circ } }}$, $M_{21}^{{60^{^\circ } }}$, $M_{33}^{{120^{^\circ } }}$ and $M_{44}^{{120^{^\circ } }}$ increase with increasing cell diameter, whereas $M_{22}^{{120^{^\circ } }}$ decreases. Therefore, these MM elements are polarization indicators sensitive to changes in cell size. Purple arrows show the diagonal elements $M_{33}^{{60^{^\circ } }}$ and $M_{44}^{{60^{^\circ } }}$ increasing with refractive index across diameters, suggesting their sensitivity to RBC refractive index. In our previous study, numerical calculations of standard particles based on Mie theory have shown that the diagonal elements of the 60° MM are related to the refractive index of the particles [31]. However, since physiologic or pathological refractive index changes in RBCs are small, MM responses are less pronounced than in particles with broader refractive index ranges.

**Fig. 4:**
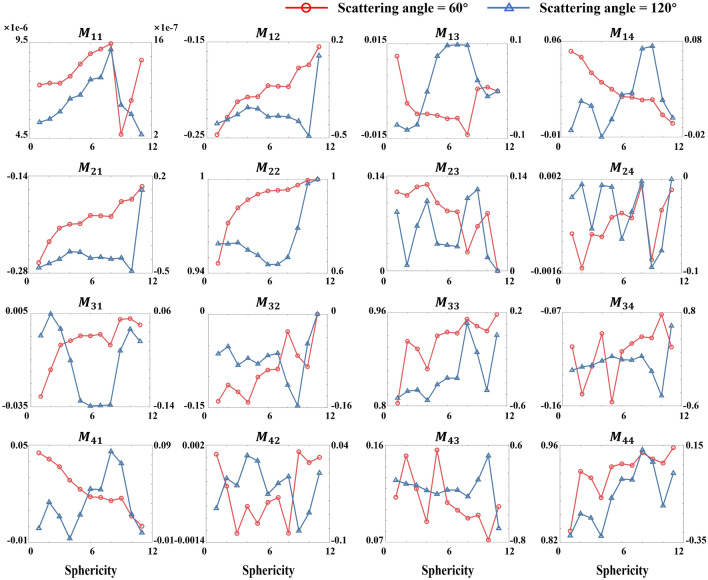
Line plot of MM at 60° and 120° for RBC models with different shapes

We next simulate varied RBC shapes to assess their impact on polarization scattering. Figure 4 presents the MM corresponding to different sphericity and concavity at 60° and 120°. Although reciprocity symmetry persists, non-diagonal elements show angle-dependent variation patterns. The transition from a flat biconcave disk to a sphere causes noticeable fluctuations in backward-scattering elements, while some forward-scattering elements show a monotonic trend. Figure 4 indicates $M_{12}^{{60^{^\circ } }}$, $M_{21}^{{60^{^\circ } }}$ and $M_{22}^{{60^{^\circ } }}$ increasing during spheroidization, while $M_{14}^{{60^{^\circ } }}$ and $M_{41}^{{60^{^\circ } }}$ decrease. Therefore, these five MM elements can serve as key indices sensitive to changes in RBC shape.

**Fig. 5:**
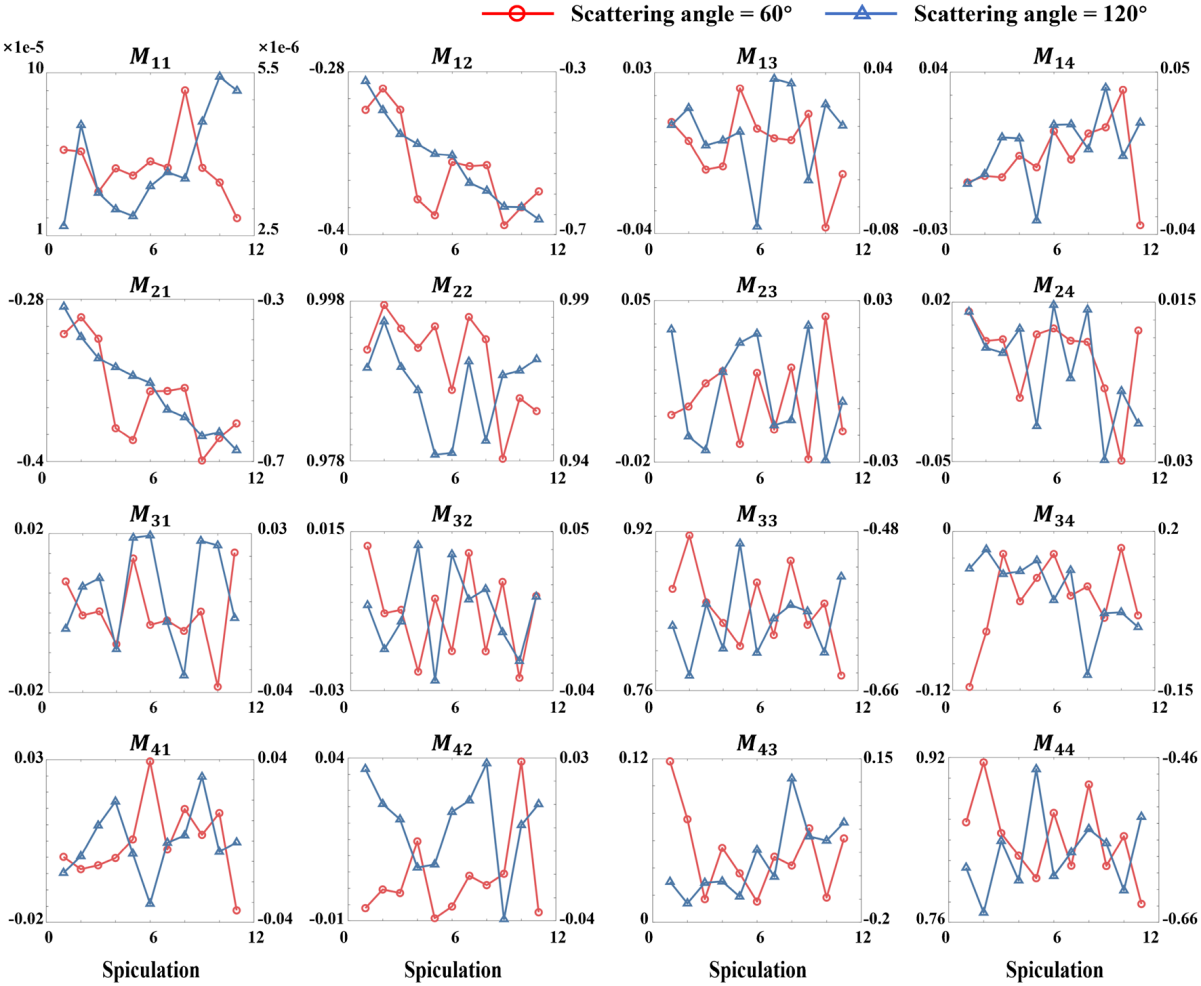
Line plot of MM at 60° and 120° for RBC models with different degrees of spiculation

To further investigate the effect of spiculated deformation on RBC MM, we use NMDDA to calculate spherical models with different degrees of surface protrusions. Figure 5 shows that randomly adding conical protrusions to the model surface disrupts both the axial symmetry of the spherical structure and the reciprocity symmetry of non-diagonal MM elements. This disruption is most pronounced for elements with smaller absolute values (e.g., $M_{13}$ and $M_{14}$). Conversely, due to the small size of protrusions relative to the cell scale, elements with larger absolute values (e.g., $M_{12}$, $M_{21}$, $M_{34}$ and $M_{43}$) retain greater symmetry. Moreover, as protrusion size increases, MM elements at 60° exhibit complex fluctuations, rendering them unsuitable deformation indicators. In contrast, the monotonic trends of $M_{12}^{{120^{^\circ } }}$ and $M_{21}^{{120^{^\circ } }}$ suggest their utility as sensitive polarization indicators for RBC spiculation.

### Polarization feature parameters (PFPs)

Although the MM comprehensively describes RBC polarization properties, its elements lack direct correspondence to specific physical properties. Therefore, we apply a feature fusion method to extract PFPs with cear physical meanings from the MM elements.

For a uniform spherical scatterer, the MM’s upper-left elements exhibit local symmetry ($$M_{11} = M_{22}$$ and $$M_{12} = M_{21}$$). Under horizontal polarization incidence ($$\overrightarrow {{\varvec{S}}}_{{{\varvec{in}}}} = [1,1,0,0]$$$$^{\text{T}}$$), the scattered Stokes vector $$\overrightarrow {{\varvec{S}}}_{{{\varvec{out}}}} = \left[ {M_{11} + M_{12} ,M_{12} + M_{11} ,0,0} \right]^{\text{T}}$$ yields equal $$s_{0}$$ and $$s_{1}$$ components. However, when RBCs transition from a biconcave disc to a sphere, their anisotropic shape disrupts this symmetry, altering the $$s_{1}$$ component changes to $$M_{12} + M_{22}$$. The variation in $$s_{1}$$ defines the morphological parameter $$K_{1}$$, as shown in Eq. (5). When $$K_{1}$$ approaches 0, the RBC shape is closer to a sphere.

5 $$K_{1} = \frac{{s_{1} - s_{1}^{\prime } }}{{s_{1} }} = \frac{{1 - M_{22}^{{60^{^\circ } }} }}{{1 + M_{12}^{{60^{^\circ } }} }}.$$

Similarly, under left circular polarization incidence ($\overrightarrow {{\varvec{S}}}_{{{\varvec{in}}}} = [1,0,0, - 1]^{\text{T}}$), the variation *s*_0_ in the component yields another morphological parameter $K_{2}$ as presented in Eq. (6).

6 $$K_{2} = \frac{{s_{0}^{\prime } - s_{0} }}{{s_{0}^{\prime } }} = \frac{{M_{14}^{{60^{^\circ } }} }}{{1 + M_{14}^{{60^{^\circ } }} }}.$$

Moreover, to characterize RBC size, refractive index, and membrane spiculation, the elements on the upper left edge (such as $M_{12}$ and $M_{21}$) or the diagonal elements (such as $M_{22}$, $M_{33}$ and $M_{44}$) were linearly combined to generate the linear orthogonal polarization attenuation differential index *LE* and the depolarization parameter *T*, expressed by Eqs. (7)–(10).

7 $$LE_{60} = \frac{{M_{12}^{{60^{^\circ } }} + M_{21}^{{60^{^\circ } }} }}{2},$$

8 $$LE_{120} = \frac{{M_{12}^{{{12}0^{^\circ } }} + M_{21}^{{120^{^\circ } }} }}{2},$$

9 $$T_{60} = \frac{{M_{33}^{{60^{^\circ } }} + M_{44}^{{60^{^\circ } }} }}{2},$$

10 $$T_{120} = \frac{{ - M_{22}^{{120^{^\circ } }} + M_{33}^{{120^{^\circ } }} + M_{44}^{{120^{^\circ } }} }}{3}.$$

### Polarization measurement of abnormal RBC deformation under experimental control

To validate the PFPs extracted from simulations for characterizing deformation-induced polarization scattering, we experimentally examined three RBC deformation types.

#### Osmotic stress

**Fig. 6:**
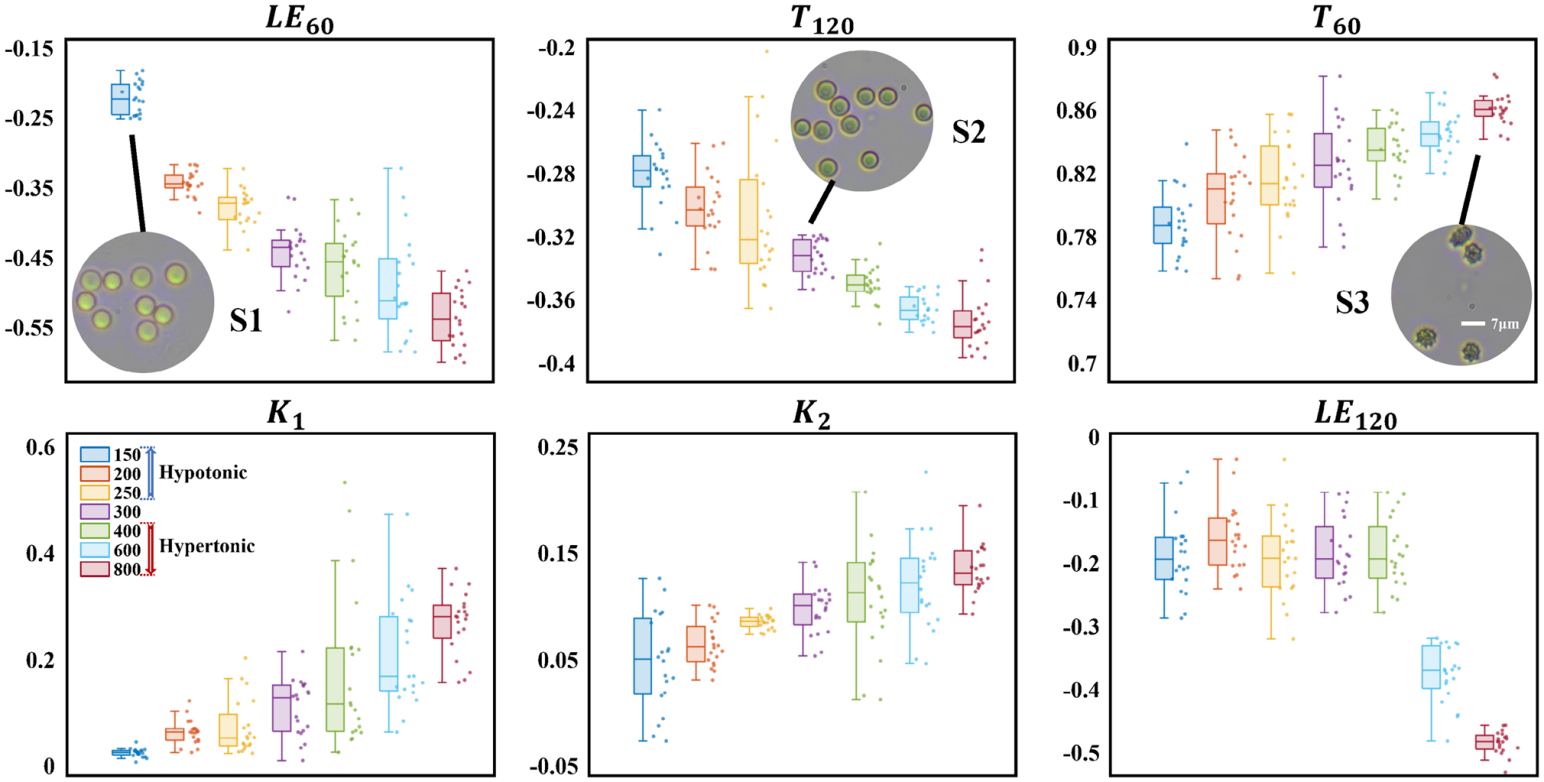
Magnitude and distribution of PFPs for RBCs at seven osmotic pressures. The subfigures show optical microscopy images of RBCs (S1), normal RBCs (S2), and acanthocytes (S3)

Transmembrane osmotic imbalances drive water movement, altering cell shape, volume, and refractive index via hemoglobin concentration changes. Using DMMP at 60° and 120°, we measure individual RBCs under varying NaCl concentrations and extracted PFPs, as shown in Fig. 6. Each box plot summarizes 500 cells.

Under hypotonic conditions (150–300 mOsm/L), water absorption increases RBC volume, elevating depolarization parameter $T_{120}$ and the linear extinction parameter $LE_{60}$ due to larger scatterer size. Concurrently, the increased cell volume leads to hemoglobin dilution, reducing the refractive index and thus decreasing $T_{60}$. Declining shape parameters $K_{1}$ and $K_{2}$ indicate reduced concavity and a transition from biconcave discs (subfigure S2) to sub-spherical shapes (subfigure S1).

Under hypertonic conditions (300–800 mOsm/L), water efflux shrinks RBCs and concentrates hemoglobin, increasing the refractive index. The corresponding polarization responses are decrease of $T_{120}$ and $LE_{60}$ while increase of $T_{60}$. Increased $K_{1}$ and $K_{2}$ reflect deeper concavity and thinner peripheral membranes. When osmolarity rises above 600 mOsm/L, membrane spiculation occurs as outer lobes expand relative to inner lobes (subfigure S3) [40]. $LE_{120}$ remains stable from 150 to 400 mOsm/L, indicating smooth membranes without spicules. However, a significant $LE_{120}$ drop above 600 mOsm/L signals RBC spiculated deformation.

#### Oxidative stress

**Fig. 7:**
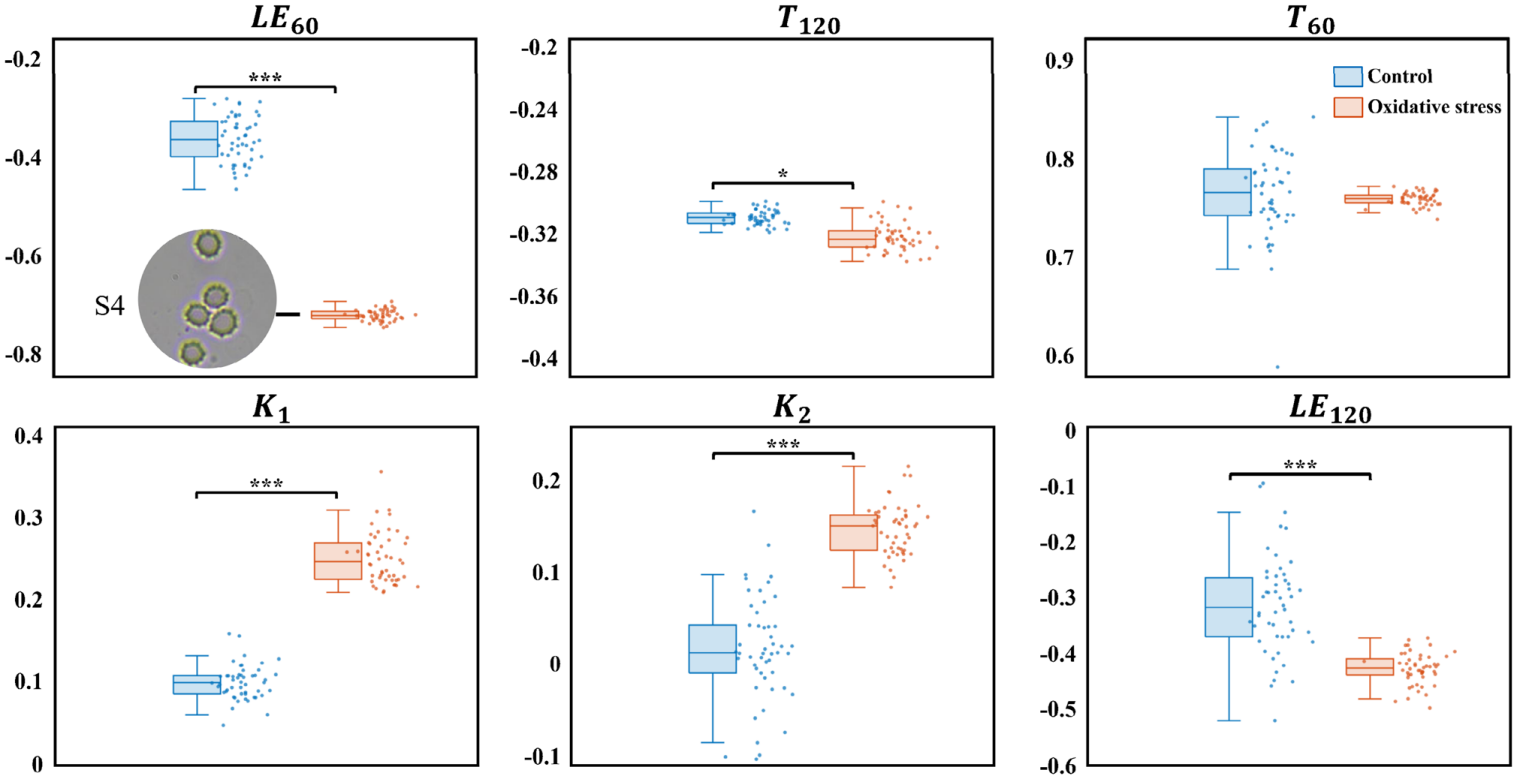
Magnitude and distribution of PFPs for the H_2_O_2_ treated group and PBS control group. S4 is an optical microscopy image of acanthocytes under oxidative stress

Oxidative stress triggers protein cross-linking and cytoskeletal remodeling, generating uneven membrane tension that promotes RBC spiculation [41]. Optical microscopy (40X) confirms spiculated deformation (subfigure S4). Figure 7 compares PFP distributions between H_2_O_2_-treated and PBS control groups.

H_2_O_2_-treated RBCs exhibit significantly increased $K_{1}$ and $K_{2}$ but decreased $LE_{120}$ compared to the control group ($p < 0.001$, *t*-test), implying ROS-driven transformation from biconcave discs to spiculated shapes with reduced sphericity and global flattening. These observations align with findings reported by Yang et al. [42].

Conversely, $T_{120}$ from the H_2_O_2_-treated group decreases slightly ($p < 0.05$), whereas $T_{60}$ is unaffected. This implies minimal oxidative impact on RBC size or refractive index. The significant $LE_{60}$ variation thus primarily stems from morphological changes rather than size.

#### pH stress

Blood pH normally ranges from 7.35 to 7.45. Abnormal pH induces hemoglobin conformational changes and cytoskeletal reorganization, significantly altering RBC morphology.

RBCs retain biconcave discs within a pH range from 7.2 to 7.6. In contrast, acidosis (pH 6.8) causes RBC swelling and reduced concavity, whereas alkalosis (pH 8.0) induces RBC shrinkage, concavity preservation, and marginal spiculation, as shown in subfigure S5 and subfigure S6.

**Fig. 8:**
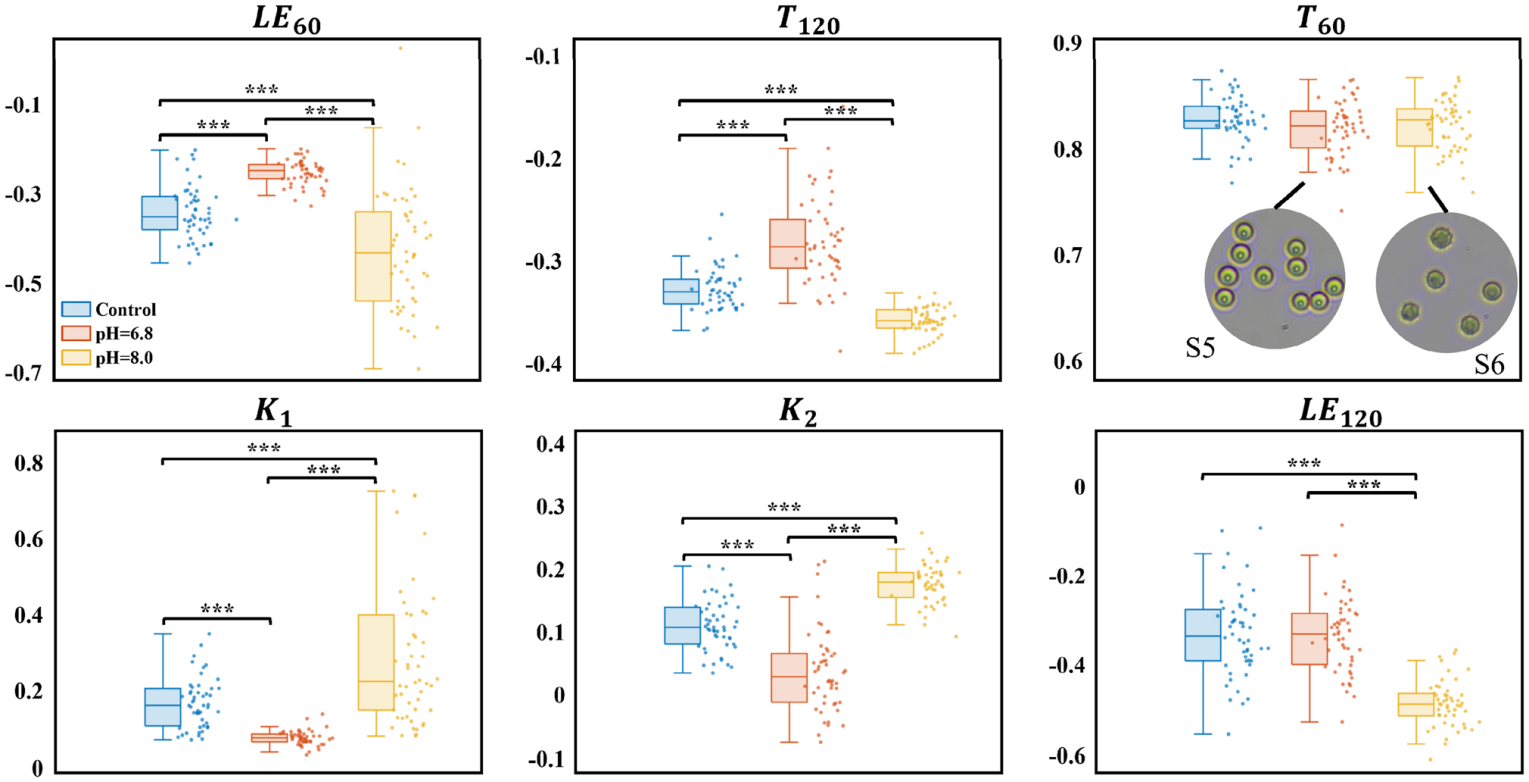
Magnitude and distribution of PFPs for RBCs in acidic, alkaline, and normal pH conditions. The subfigures show optical microscopy images of RBCs (S5) at pH = 6.8 and acanthocytes (S6) at pH = 8

As shown in Fig. 8, DMMP measurement reveals that acidosis significantly decreases $K_{1}$ and $K_{2}$ but increases $T_{120}$ and $LE_{60}$ versus physiologic pH, indicating reduced concavity, enhanced sphericity, and larger size. Stable $LE_{120}$ confirms smooth membranes. Conversely, alkalosis elevates $K_{1}$ and $K_{2}$ but reduces $T_{120}$ and $LE_{60}$, signaling RBC shrinkage and diminished sphericity. Significantly increased $LE_{120}$ further demonstrates the formation of membrane spiculation. Additionally, $T_{60}$ remains consistent across three pH control groups, confirming negligible pH impact on intracellular refractive index.

### Assessment of the proportion of abnormal RBCs in mixed RBC suspension

To further validate PFP reliability for characterizing RBC deformation, we employ DMMP to measure mixed RBC suspensions composed of normal, spherical, and spiculated RBCs. The PFPs or MM elements are used as input features for the RF classifier to quantify RBC type proportions. The training set consists of MM data from three monodisperse RBC suspensions obtained by the osmotic stress regulation, with each cell type containing 2000 data points. Each data point includes a feature vector of 30 MM elements ($M_{11}$ was used only for normalization, not for prediction) or six PFPs. The test set is the actual measurement data from the mixed RBC suspension.

**Fig. 9:**
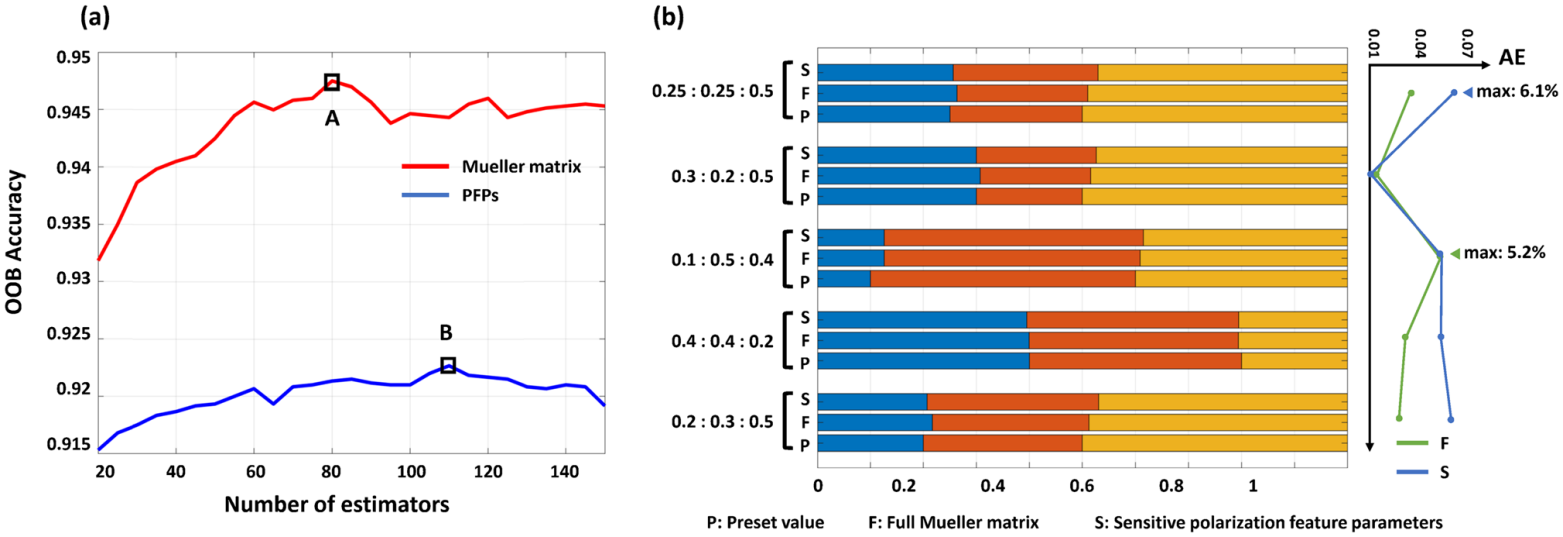
**a** OOB accuracy graph for RF estimation with MM elements or PFPs as inputs. **b** Results and absolute errors of the proportional analysis for mixed RBC suspensions

The number of decision trees critically influences RF performance, and its optimization can improve accuracy, reduce complexity, and enhance generalization. Out-of-bag (OOB) data are used to determine the optimal number of decision trees, as shown in Fig. 9a. When the number of decision trees is small, the model’s OOB accuracy can be improved with the increasing number. Then, the model’s classification performance gradually stabilizes. Specifically, Specifically, optimal tree number is 80 (A) for MM elements and 110 (B) for PFPs.

Then, the trained RF classifier predicts RBC types in the test set using either a 30-dimensional MM vector or a 6-dimensional PFP vector, and the prediction error is quantified via absolution error (*AE*):

11 $$AE = \sum\limits_{i = 1}^{3} {\left| {T_{i} - P_{i} } \right|},$$

where $T_{i}$ is the true proportion and $P_{i}$ is the predicted proportion for cell type *i*.

Figure 9b shows that both models achieve high accuracy for abnormal RBC identification, with a maximum of 5.2% for the MM element-based model and 6.1% for the PFP-based model. Though the MM-based RF classifier showed marginally better accuracy, PFPs provide superior physical interpretability for deformation mechanisms, which validates PFPs’ utility in blood abnormality screening.

## Conclusions

In this study, we integrate the DMMP technique with RBC polarization scattering simulations to extract specific MM elements. These elements enable the identification and characterization of subtle changes in RBC size and morphology. Three typical RBC deformation types, including sphering, edge flattening, and surface spiculation, are modeled, and their polarization light scattering characteristics are simulated using the DDA method. We establish correlations between microscopic cellular physical property changes and polarization responses, quantitatively evaluating alterations in RBC size, refractive index, shape, and surface spicules using full polarization measurements.

Through a feature fusion method, we extract six PFPs with distinct physical interpretations that reflect combined size and shape changes: shape parameters $K_{1}$ and $K_{2}$, size parameter $T_{120}$, refractive index parameter $T_{60}$, surface parameter $LE_{120}$, and composite $LE_{60}$.

To verify PFPs’ effectiveness in characterizing RBC deformation, we conduct three types of stress experiments that induce abnormal cell deformation. The results show significant differences in PFPs between the experimental and control groups ($p < 0.001$), which is consistent with optical microscopy observations and prior studies. Furthermore, an RF classifier processed PFPs from mixed RBC suspensions, successfully determining proportions of abnormally deformed cells with a maximum *AE* of 6.1%. These results demonstrate the robustness of PFPs for identifying abnormal RBC deformation. Compared to the full MM, PFPs offer more intuitive physical interpretations of polarization responses linked to deformation. This method shows significant potential for rapid, high-throughput, label-free analysis of RBC-related pathology at the single-cell level, providing a valuable new approach for diagnosing blood disorders.

## Data Availability

The data that support the findings of this study are available from the corresponding author, upon reasonable request.
